# Domestic Intelligence

**Published:** 1800-04

**Authors:** 


					Miscellaneous and Domestic Intelligence. 377
Domefiic Intelligence.
Dr. Batty will begin his ufual Courfe of Le&ures on the The-
ory and Pra&ice of Midwifery, and on the Difeafes of Women and
Children, on Monday, April 7th, at half paft ten o'clock in the
morning, at his houfe, No. 6, Great Marlborough Street.
Dr. Willich has in the prefs, the third Edition of his " Lec
tures on Diet and Regimen." ? K
Numb. XIV. Ccc CRITICAL
- \ .

				

